# Protecting organ donation and transplantation programmes from regulatory uncertainty: an urgent appeal to the European Parliament, Council and Commission

**DOI:** 10.1016/j.lanepe.2026.101650

**Published:** 2026-03-21

**Authors:** Natividad Cuende, Ander Izeta, Beatriz Domínguez-Gil

**Affiliations:** a Andalusian Transplant Coordination, Servicio Andaluz de Salud, c/ Imagen 12, 41003, Sevilla, Spain; b Advanced Therapies Unit, Donostia University Hospital, Paseo Dr. Begiristain s/n, 20014, Donostia-San Sebastian, Spain; c Stem Cells and Aging Group, Biogipuzkoa Health Research Institute, Paseo Dr. Begiristain s/n, 20014, Donostia-San Sebastian, Spain; d Organización Nacional de Trasplantes, c/ Sinesio Delgado 6, pabellón 3, 28029, Madrid, Spain

Recent scientific advances in organ transplantation are poised to expand organ availability and improve post-transplant outcomes. Innovations such as enzymatic and genetic manipulation—enabled by *ex-vivo* organ perfusion—offer new hope to patients and may improve European competitiveness in the biotechnology sector, as intended by the European Commission's current proposal for a Biotech Act.^1^

However, the accompanying draft Directive amending the European Organ Directive^2^ generates regulatory uncertainty that could ultimately create significant challenges for European healthcare systems, due to conflicting legal and regulatory frameworks as well as ethical issues arising from such changes. Specifically, the amendment extends the Directive's scope to include “organ processing”, defined as “*operations involving the handling of organs, including but not limited to preservation, application of chemotherapy and surgery, performed to maintain or improve organ function prior to transplantation. The definition excludes the use of substances with a pharmacological, immunological or metabolic action where the primary aim is to treat or prevent a disease in the recipient and not to process the organ*.” This wording raises significant concerns, particularly in light of the recent recommendation by the Committee for Advanced Therapies of the European Medicines Agency to classify a viral vector intended to modify human lungs *ex-vivo* prior to transplantation, to mitigate immune-mediated rejection, as an advanced therapy medicinal product (ATMP).^3^ Such manipulations could fall outside the definition of processing, placing genetically modified human organs (MHO) beyond the scope of the Directive and opening the door to their regulation as medicines.

A major issue is how such “MHO-based therapies” could be integrated into existing donation and transplantation systems. These are currently grounded in the principles of altruism, non-remuneration for organs and equitable access. In contrasts, access to ATMPs depends on market authorisation by for-profit entities, followed by pricing negotiations, reimbursement decisions and commercial strategies.^4^ Furthermore, treating MHO as market commodities risks undermining public trust and discouraging donation. The mere prospect that an altruistically donated and subsequently modified organ could be commercialised, enabling wealthier individuals to pay for transplantation with purchased MHO, would compromise the principle of reciprocity underpinning donation systems. Moreover, it could ultimately lead to the breakdown of donation and transplantation programmes. Additionally, and since MHO originate from altruistic donation, their commercialisation would conflict with foundational ethical principles endorsed by the World Health Organisation, the Council of Europe and the EU Charter of Fundamental Rights. This includes the prohibition on making the human body or its parts a source of financial gain.^5^

Of note, the experience of the last two decades with ATMPs suggests that shifting therapies from the transplantation to the pharmaceutical regulation may also impose unsustainable financial burdens on public health systems, limit and create unequal patient access, and result in significant disparities across EU Member States.^6^, ^7^, ^8^, ^9^, ^10^

Possibly the main argument to switch legal framework and increase regulatory oversight on MHO is the fact that they pose additional biological risks as compared to non-modified organs. These risks, together with the quality, safety, functionality and effectiveness of the MHO, must be assessed according to rigorous standards proportionate to the risk level. To reconcile innovation with ethical integrity, we put forward a model that retains organs—regardless of the manipulations applied or their therapeutic or preventive use—within the transplant regulatory framework (Fig. 1). We propose to distinguish between the assessment and the authorisation procedures, ensuring comprehensive collaboration among the relevant authorities, and to differentiate organ ownership from ownership of the technology to manipulate organs. We also advocate strengthening evaluation standards through close collaboration between medicines and transplant authorities.

**Fig. 1:**
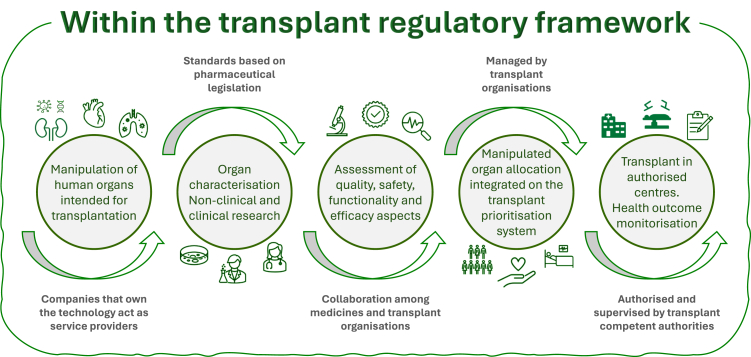
**Main elements of a model to assess and authorise the transplantation of modified human organs within the transplant regulatory framework and the role of stakeholders.** The model is based on the following key aspects: 1. Integrate the human organ donation process within official programmes, with donor selection based on scientific and ethical criteria approved by the competent authorities responsible for organ donation and transplantation. 2. Distinguish ownership of ORGANS from ownership of the TECHNOLOGY used for their manipulation. 3. Separate ASSESSMENT (pharmaceutical based standards may apply, evaluated collaboratively by medicines and transplant authorities) from AUTHORISATION (by transplant authorities). 4. Integrate modified human organs into the organ allocation system, with eligible patients registered on “official” waiting lists under the supervision of transplant organisations. 5. Ensure that human organs, regardless of manipulation or indication, remain regulated under the transplant framework and are not commodified—essential to maintain the current organ donation and transplantation system.

Two additional key elements must be addressed to ensure the proper integration of MHO into donation and transplantation systems without compromising their fundamental principles. First, donor selection for organs intended for manipulation must follow scientific and ethical criteria approved in advance by the competent authorities in transplantation, and the donation process must remain fully embedded within official donation and transplantation programmes. Second, MHO must be incorporated into the organ allocation system. Accordingly, all patients eligible for transplantation should be registered on “official” waiting lists under the supervision of the competent authorities for organ transplantation.

Only by ensuring that manipulated human organs remain regulated as transplants can Europe advance scientifically without undermining the ethical foundations that make its transplantation system a global benchmark.

## Contributors

NC: Conceptualisation, Data curation, Visualisation, Writing—original draft.

AI: Conceptualisation, Data curation, Writing—review & editing.

BDG: Conceptualisation, Data curation, Writing—review & editing.

## Declaration of interests

The authors declare no competing interests.
